# Predictive Model of Lake Photic Zone Temperature Across the Conterminous United States

**DOI:** 10.3389/fenvs.2021.707874

**Published:** 2021-10-18

**Authors:** B. J. Kreakie, S. D. Shivers, J. W. Hollister, W. B. Milstead

**Affiliations:** US Environmental Protection Agency, Office of Research and Development, Atlantic Coastal Environmental Sciences Division (ACESD), Narragansett, Ri, United States

**Keywords:** random forest, US EPA National Lakes Assessment, limnology, water temperature, warming lakes, photic zone temperature

## Abstract

As the average global air temperature increases, lake surface temperatures are also increasing globally. The influence of this increased temperature is known to impact lake ecosystems across local to broad scales. Warming lake temperature is linked to disruptions in trophic linkages, changes in thermal stratification, and cyanobacteria bloom dynamics. Thus, comprehending broad trends in lake temperature is important to understanding the changing ecology of lakes and the potential human health impacts of these changes. To help address this, we developed a simple yet robust random forest model of lake photic zone temperature using the 2007 and 2012 United States Environmental Protection Agency’s National Lakes Assessment data for the conterminous United States. The final model has a root mean square error of 1.48°C and an adjusted R^2^ of 0.88; the final model included 2,282 total samples. The sampling date, that day’s average ambient air temperature and longitude are the most important variables impacting the final model’s accuracy. The final model also included 30-days average temperature, elevation, latitude, lake area, and lake shoreline length. Given the importance of temperature to a lake ecosystem, this model can be a valuable tool for researchers and lake resource managers. Daily predicted lake photic zone temperature for all lakes in the conterminous US can now be estimated based on basic ambient temperature and location information.

## INTRODUCTION

During a time of unprecedented environmental and climatic variability, lakes can serve as sentinels and integrators in a changing world (Schindler 2009; Williamson et al., 2009). As the average global air temperature increases (0.15–0.20°C per decade between 1975 and 2009) (Hansen et al., 2010), surface temperatures of lakes are also increasing globally (0.34°C per decade from 1985 to 2009) (O’Reilly et al., 2015).

The influence of this increased temperature touches all biotic and abiotic components of lentic ecosystems. For example, warming lakes are linked to a disruption in trophic linkages between phytoplankton and zooplankton (Winder and Schindler 2004). Specifically, Winder and Schindler (2004) report a spring diatom bloom occurring 20 days earlier and a long-term decline in *Daphnia* populations in Lake Washington. Warming has also been shown to result in changes in thermal stratification across different lake types (O’Reilly et al., 2003; Verburg et al., 2003; Cohen et al., 2016; Michelutti et al., 2016; Hampton et al., 2018). In particular, lakes that typically stratify can exhibit a stratification strengthening as well as a reduction in the depth of the thermocline or previously polymictic lakes can begin to stratify as a result of warming lake temperatures. Temperature, in addition to nutrients, is a key driver of cyanobacteria bloom dynamics (Paerl and Paul 2012). During periods of higher temperature, cyanobacteria species dominate the phytoplankton community (Peperzak 2003; O’neil et al., 2012; Lürling et al., 2013). As lake temperature increases (typically above 25°C), cyanobacteria have a competitive advantage over phytoplankton and can proliferate quickly (Paerl and Huisman 2008). Moreover, experimentally enhanced water temperatures yielded significantly increased growth rates of toxic *Microcystis*, but not the non-toxic strains (Davis et al., 2009). Thus, our ability to understand and predict toxic cyanobacteria blooms will be deeply dependent on our ability to forecast lake temperature.

Ultimately, temperature changes will greatly impact every aspect of lake ecology and lake resource management. Specifically, and the focus of our work, the temperature changes in the near-surface area (photic zone) where the majority of photosynthesis occurs will have exponential impacts on lake ecosystems. Because of the ecological significance, it is not surprising that modelling lake photic zone temperature has been broadly investigated. Models typically vary in number of lakes studied, complexity of modelling approach, and study interval. Modelling efforts include attempts to model a single lake over relatively small (e.g., hourly) time intervals (Peeters et al., 2002; Saeed et al., 2016; Zhong et al., 2016). Additionally, there are numerous studies that model temperature for a small number of lakes (14–17 lakes) while attempting to limit the number of predictor variables (Matuszek and Shuter 1996; Livingstone and Lotter 1998; Kettle et al., 2004; Toffolon et al., 2014; Piccolroaz 2016). In these efforts, air temperature and lake size are often the only selected predictor variables. Others have aimed at modelling and measuring lake temperature across large spatial extents for large number of lakes (291–388 lakes) (O’Reilly et al., 2015; Minns et al., 2017; Wan et al., 2017). These past attempts use a wide variety of approaches and have been rather successful at modelling lake photic zone temperature. One limitation, however, is the majority of these lakes are large and few studies document or model smaller lakes (although see the following for examples of larger spatial extent studies that focus on small lakes: Fang and Stehan 1999, Kettle et al., 2004, Read et al., 2014, and Winslow et al., 2015). Considering that the number and importance of small lakes (<1 km^2^) has been historically underestimated (Downing et al., 2006; Winslow et al., 2014), it is important to explicitly include smaller waterbodies into temperature related research.

In spite of the need to understand near-surface temperature for all lakes across a large spatial extent, there have been several challenges that have slowed our progress. Modelling lake temperature requires a large amount of data, and therefore study lakes are often selected opportunistically, which may introduce a spatial bias. Frequently, the study lakes have a high regional resource value (e.g., Laurentian Great Lakes) and commonly have an extensive monitoring history due to the vested interest of the public. When modelling efforts do attempt to cross large spatial extents, these efforts often rely on satellite data. While these models predict over large areas, they are restricted by the size of the lake captured by satellite (typically 3 km^2^ for 1 km Moderate Resolution Imaging Spectroradiometer (MODIS) pixels).

Our modelling effort takes advantage of the relatively recent availability of broad scale field data for lakes and uses the United States Environmental Protection Agency’s (US EPA’s) National Lakes Assessment (NLA). The NLA is a stratified random sample of all lakes in the conterminous United States repeated every 5 years beginning in 2007. Even though this is a large effort involving numerous agencies, the sampling methods are standardized and have a comprehensive quality assurance plan. The uniqueness of this data set allows us to build a robust lake photic zone temperature model for all US lakes. By using lakes across the US (i.e., at a large spatial extent), we included lakes with different morphologies and in different climates with diverse geologies and surrounding landscapes. Additionally, small lakes were well represented with more than 50% of the lakes in the 2012 survey less than 0.5 km^2^. Our modelling approach, random forest, is well adapted to fully utilize these data. Random forest is a relatively novel machine learning algorithm with convergences based on the parameter strength and not deterred by the noise (Biau 2012).

The main goals of this work are to 1) develop a simple yet robust lake photic zone temperature model and 2) develop a model that is applicable to all lakes in the conterminous US that captures key drivers of near-surface lake temperature. Additionally, it is our practice to conduct our work as openly as is feasible. Towards that end we provide access to all code and data used to develop these models with the active repository available at https://github.com/usepa/lake_photic_zone.

## METHODS

### Data

**FIGURE 1 ∣:**
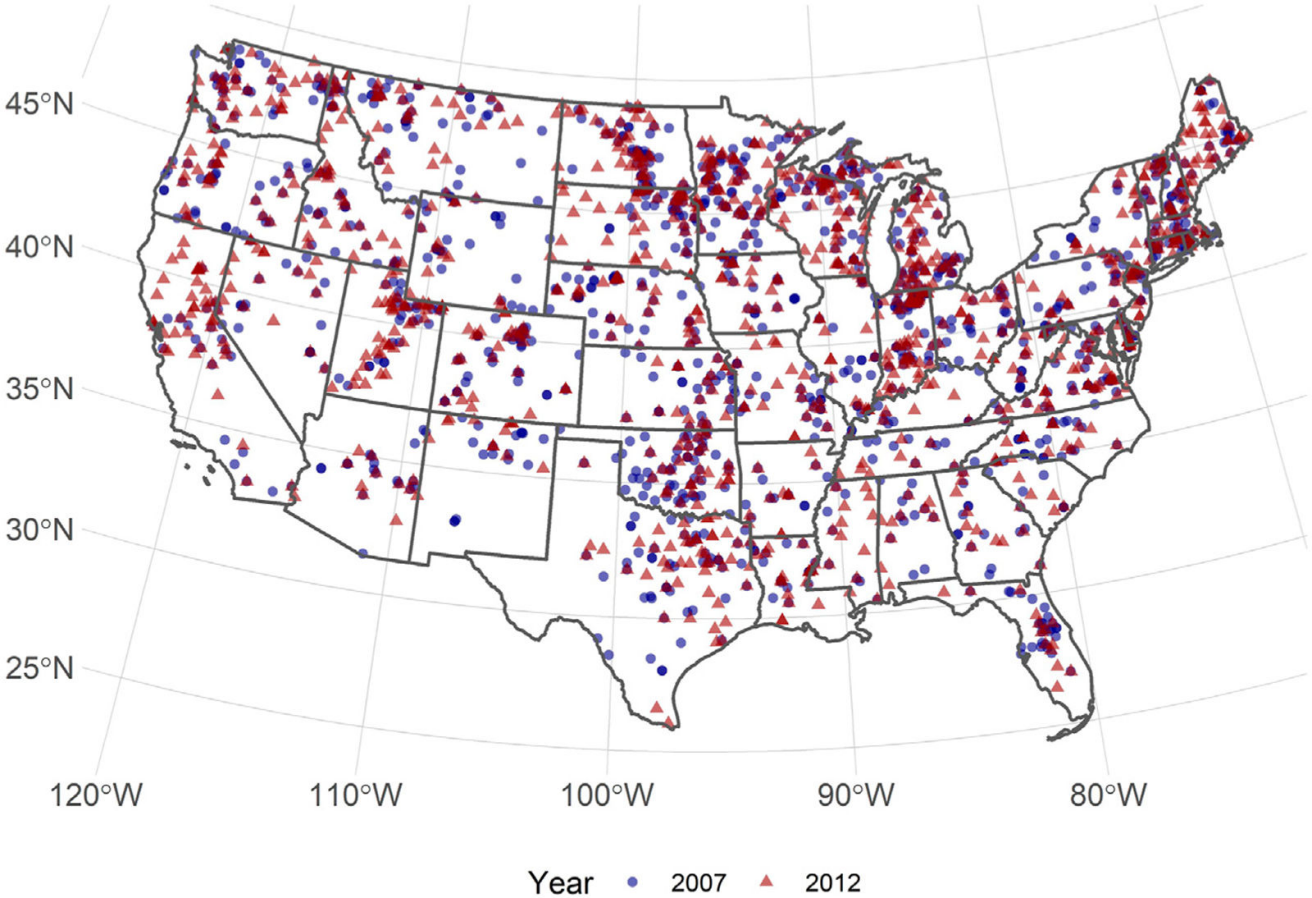
Map of 2007 and 2012 US EPA National Lakes Assessment.

We relied on the *in situ* temperature data provided in the US EPA’s National Lakes Assessment (US Environmental Protection Agency 2009, 2016). The NLA is a generalized random tessellation stratified sample of lakes (greater than 1 ha) across the US (US EPA 2011). The lakes are sampled between 01 May and 30 September, and a few individual lakes (~10%) are resampled during that sample period (Hart and Bell 2015). NLA sampling took place in 2007, 2012, and 2017. This research effort used the 2007 and 2012 sample years. The 2017 data are currently undergoing quality control before being released to the public. For both sample years, we have included lakes across the conterminous US excluding the Laurentian Great Lakes (Figure 1). For each sampled lake, we used the mean temperature for all sampled depths of less than 2 m. While the photic zone encompasses the water column depth within which photosynthesis is primarily occurring due to the penetration of light (Wetzel 2001), we generalized to 2 m in order to model across a large spatial extent. Adapting photic zone depth by lake and through time are beyond the data available to us.

We included numerous predictor variables that are hypothesized to impact lake photic zone. As a proxy for directly measured ambient air temperature, we used the PRISM AN81 d data set (PRISM Climate Group, Oregon State University, http://prism.oregonstate.edu, created Nov 07, 2018). This data set provides interpolated daily temperature estimates (mean, maximum, and minimum) for 4 km grids in the conterminous US from 1981 to the present (see: http://www.prism.oregonstate.edu/documents/PRISM_datasets.pdf). PRISM takes advantage of measured climate variables to interpolate point data to spatially defined grids using regression techniques and expert knowledge (Daly et al., 2008). For our study, we used the *prism* R package to download the mean daily temperatures for the PRISM grid cells corresponding to the centroids of all NLA lakes included in this study.

In addition to climate, there are other factors that are thought to impact lake temperature (e.g., surrounding land use, lake depth, size and configuration, and elevation) (See Supplementary Table S1 in Supplemental Material for full list of initial model variables). To test for the relative importance of lake morphometry and surrounding landscape, we used the R packages *lakemorpho* to calculate a suite of lake morphometry metrics and *elevatr* to access digital elevation models for each lake (Hollister and Stachelek 2017; Hollister and Tarak Shah 2017). Non-point run-off from impervious surface into lakes supplies potentially large volumes of warm water (Brabec et al., 2002). Therefore, we used the National Land Cover Database (NLCD) as a source for land cover data (Homer et al., 2004; Homer et al., 2012) and calculated the percent impervious surface of a 3,000 m buffer for each lake. Lakes with partial buffers falling outside the US were excluded. The 3,000 m buffer was selected as an intermediate scale that represents an area larger than the immediate surroundings but does not encompass the entire basin (Hollister et al., 2016). To test the relative influence of both short and long-term temperature, we derived several measures for a lake’s local air temperature. Mean air temperatures for day of and the day before the sample date were extracted directly from the PRISM data. To understand longer term influences, we calculated average mean air temperatures for periods 3, 7, and 30 days prior to the sample date. The NLA samples throughout the growing season. Sample date was included to capture the effect of warming that occurs during the growing season. See Table 1 for summary of predictor variables selected for the final model.

### Random Forest Modelling

Random forest modelling was used to not only develop a predictive model of photic zone temperature, but also used as a means of variable selection and to calculate relative variable importance. Random forest is a machine learning method that builds a consensus prediction from the assemblage of multiple tree models (here specifically 10,000 trees for the final model and 1,000 trees for the variable selection models). Each individual tree model was constructed from a bootstrapped subset of the full data set; each sample was drawn at random which results in approximately 2/3 of the data set selected into the training set. Also, a subset of all predictor variables was selected for each tree (p/3 where p is the number of variables). All random forest modelling was conducted in R v 4.0.3 (R Core Team 2020) with the *randomForest* R package (Liaw and Wiener 2002). Model performance was reported as mean square error and adjusted R^2^. See Breiman 2001 for more detailed methods.

Random forest does not require that users reduce the number of predictor variables because the random forest algorithm prevents overfitting and is not impacted by correlated predictor variables (Cutler et al., 2007). It is unlikely that random forest models constructed with reduced numbers of predictor variables perform any better than models constructed with a full suite of available variables (Fox et al., 2017). However, reducing the number of predictor variables eases interpretation and can reduce potentially unneeded computation time while, as demonstrated in the Results, not severely impacting our prediction accuracy. Several of the climatic predictor variables were computationally intensive to create for the entire conterminous US. In order to use our final model for future forecasting or historical backcasting, we strove to create a robust predictive model while minimizing computational demands. To determine the optimal number and set of variables, we followed the variable selection method presented in Hollister et al., 2016. This variable selection method requires fitting a random forest model with the full set of variables and then ranking the variables according to each variable’s increase in mean square error (described below). Then, random forest models are iteratively fit with the sequential addition of variables based on the ranking. This allows us to see when additional variables no longer improve the fit of the model. Essentially creating a model with maximized accuracy with minimal variables. We evaluated the resultant model in several ways. First, we assessed the overall model performance with traditional measures such as root mean square error (RMSE). For random forest, this is a vector of mean square errors divided by the total number of trees. Second, we examined error by comparing the predicted versus observed temperature for all lakes. This method uses the final model to predict photic zone temperature for all lakes in the data set. In addition to these measures of overall model performance, we used percent increase of mean square error to assess variable importance. The percent increase in mean square error is a comparison between the mean square error for the model fit with the true values of a variable and a model fit with randomly permuted vector of variable values. Finally, partial dependence plots were used to visualize the partial relationship between individual variables and the response variable (Hastie et al., 2009).

## RESULTS

Our final model was constructed with 1,185 data points from the 2007 NLA and 1,097 from the 2012 NLA across the conterminous US (Figure 1).

**FIGURE 2 ∣:**
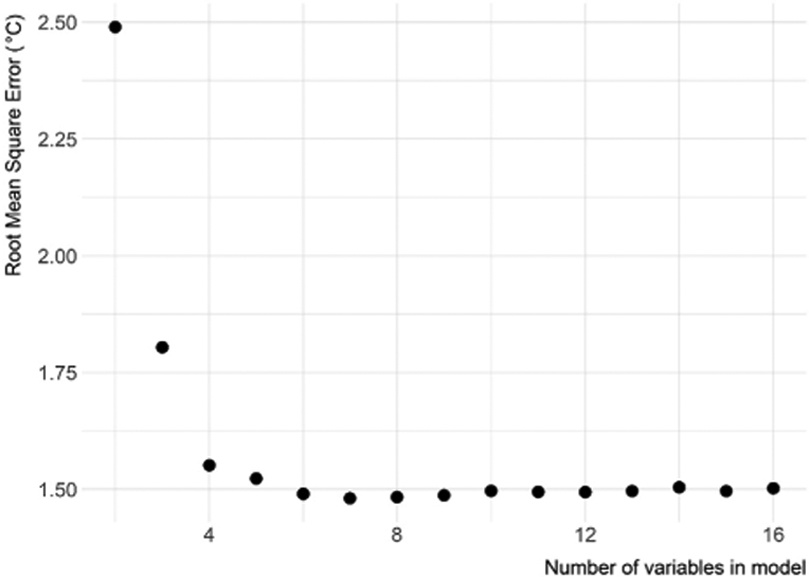
Variable selection plot for all variables. Shows root mean square error as a function of the number of variables. The variables, listed in order of initial random forest ranking, are average ambient air temperature, sample date, longitude, 30-days average temperature, elevation, latitude, shoreline length, surface area, maximum lake depth, percent impervious surface for 3,000 m buffer, maximum lake length, year, shoreline development, mean lake width, volume, and maximum lake width.

Using the average lake temperature for the upper 2 m as the response variable, we initially began the variable selection process with 16 predictor variables. The variables included were average ambient air temperature for the sample date, sample date, longitude, average ambient air temperature for 30 days preceding the sample date, elevation, latitude, length of lake shoreline, lake surface area, maximum lake depth, percent impervious surface for 3,000 m buffer, maximum lake length, year, shoreline development, mean lake width, volume, and maximum lake width (variables are listed in order of initial random forest ranking and, therefore, the order each variable was iteratively included in random forest). The variable selection process identified a reduced model with ~8 variables showing minimal model error (Figure 2). The selected variables were average ambient air temperature, sample date, longitude, 30-days average air temperature, elevation, latitude, shoreline length, and lake surface area (Table 1).

**FIGURE 3 ∣:**
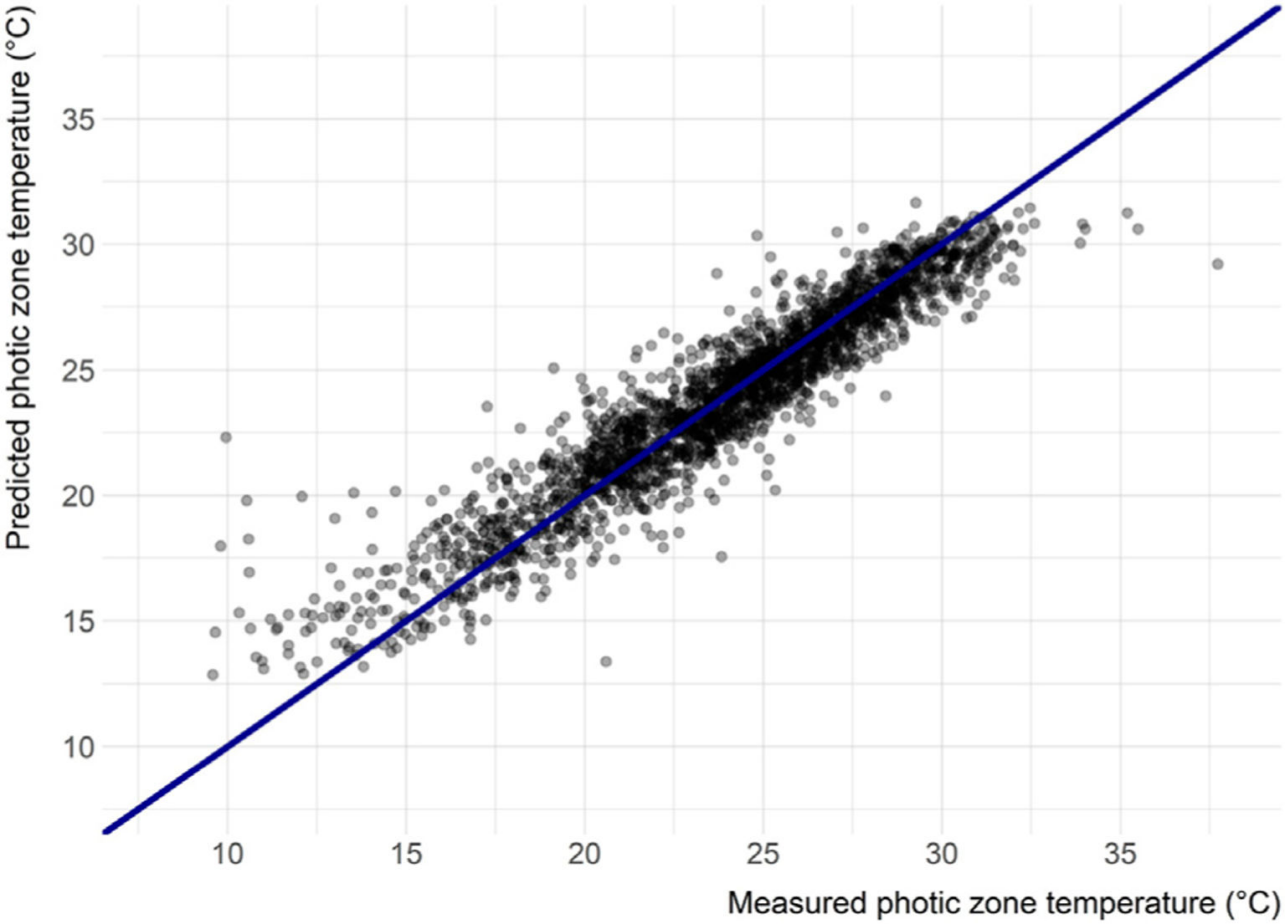
Predicted versus measured photic zone temperature (n = 2,282).

**FIGURE 4 ∣:**
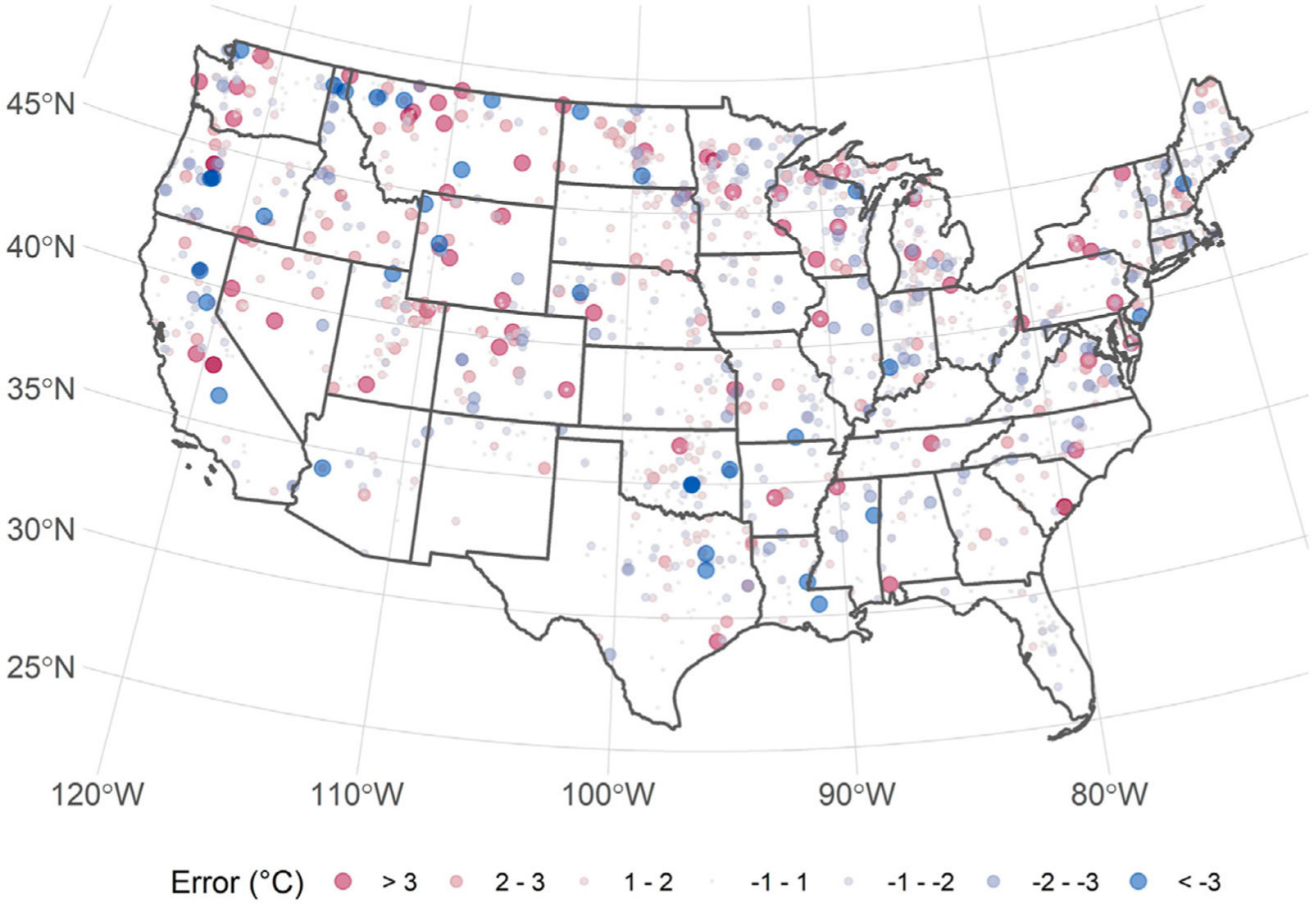
Map of predicted minus observed temperature for 2007 and 2012 National Lakes Assessment.

The final model built with the eight selected variables has a RMSE of 1.48°C and adjusted R^2^ of 0.88. The model performs well across a wide range of temperatures (Figure 3); however, at the higher and lower temperature (i.e., less than 15°C and greater than 32°C) the model does not perform as well, but lakes with growing season temperatures at these extremes were rare and represented only 3.86% of lakes sampled in the 2007 and 2012 NLA. Additionally, there is not an obvious spatial clustering of lakes with higher error (Figure 4). The spatial autocorrelation of model error overall is uninteresting (Moran’s I = 0.028, *p*-value = 0.000015) (Legendre and Fortin 1989). We also explored the correlation between each predictor variable and error; there was found no relationship [ranging from −0.076 (date) to 0.105 (average air temperature)].

**FIGURE 5 ∣:**
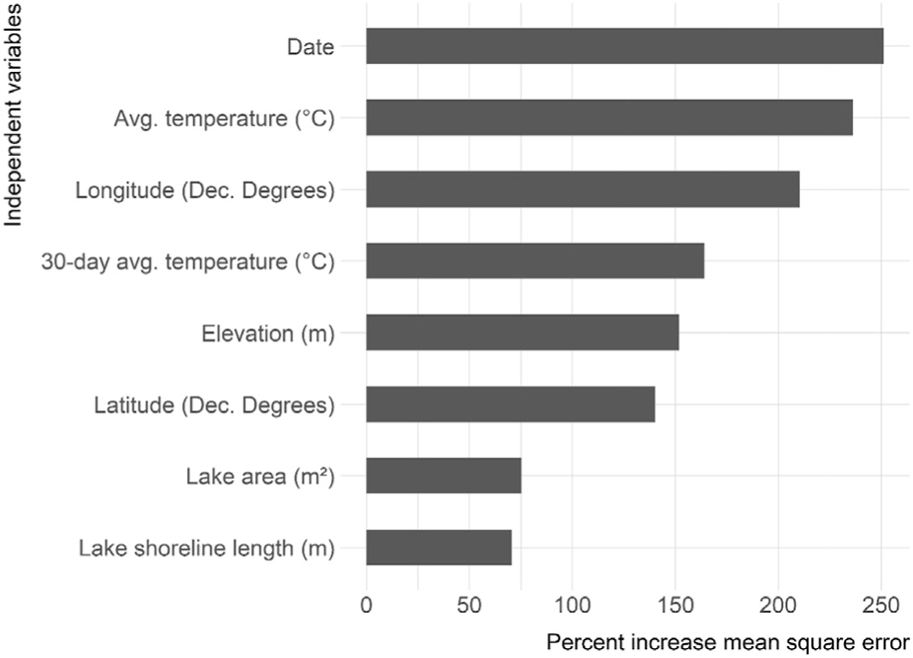
Variable importance plot for selected variables. Shows percent increase in mean square error. Higher values indicate a higher impact on overall model accuracy.

**FIGURE 6 ∣:**
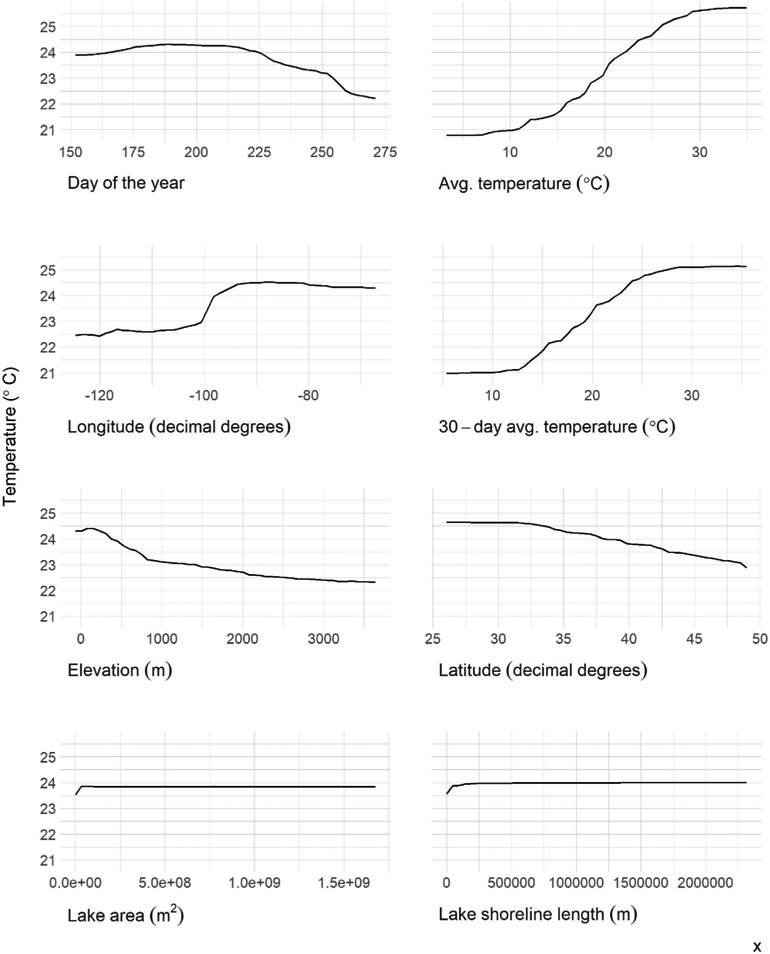
Partial dependence plots for selected variables.

The variables ranked in order of importance were date, average temperature, longitude, 30-days average temperature, elevation, latitude, surface area, and shoreline length (Figure 5). The partial dependency plots illustrate how the predicted photic zone temperature changes over the range of values for all predictor variables (Figure 6).

## DISCUSSION AND CONCLUSION

Here, we present a simple yet robust model of lake photic zone temperature using the 2007 and 2012 NLA data for the conterminous US. The final model has a RMSE of 1.48°C and an adjusted R^2^ of 0.88. Given the importance of temperature to a lake ecosystem, especially to cyanobacteria bloom dynamics (Robarts and Zohary 1987.; Paerl and Otten 2013), this model can be a valuable tool for researchers and lake resource managers. Daily predicted lake photic zone temperature for all lakes in the conterminous US can now be estimated based on basic ambient temperature and location information. Despite overall well-behaved performance, the final model is less accurate at extreme temperatures (Figure 3). As might be expected, the model underestimates temperature for relatively high temperature lakes and overestimates colder lakes. Given that each tree of the random forest is built on a subset of all sample points and the rarity the extreme temperature events, it is difficult to improve the tails of the temperature distribution. It is expected that the addition of new NLA data will improve both the overall model performance and predictions for the more rare events. Yet Figure 4 illustrates there is no spatial clustering to these errors. While we might be less certain with the predictions for extremely rare events, we are confident in the model’s ability to reliably predict across all regions of the conterminous US.

Sampling date, the sampling day’s average ambient air temperature, and longitude are the most important variables impacting the final model’s accuracy (Figure 5). The 30-days average temperature, elevation, and latitude comprise the second tier of important variables. The final tier of variable importance is lake area and shoreline length - the only morphometric variables included in the final model. Morphometry describes measures of lake form and size that have vast influence on numerous lake functions (e.g., transport and stratification) (Hakanson 2012). Given the importance of these measures, it is striking that morphometric variables did not have a larger impact on this model. A (Sharma et al., 2008) photic zone temperature model of 2,348 Canadian lakes had similar results and conclusions. This research found that near-surface temperatures were accurately predicted by large-scale climate and geographic patterns, and not lake-specific measures.

In addition to the morphometric variables, we calculated the percent impervious surface for a 3,000 m lake buffer. This measure was included based on the hypothesis that higher amounts of development and impervious surface surrounding a lake would lead to higher temperatures in lakes (Yang et al., 2019). Yet this variable was not selected in the final model. Even though the land-use variable was not selected, that does not mean development and impervious surface are not important to the local temperature. The urban-heat effect on lakes may have been adequately captured in the average ambient air temperature or alternatively lakes can potentially serve as urban-heat temperature sinks (Steeneveld et al., 2014; Cosgrove and Berkelhammer 2018). Land-use variables would be redundant or irrelevant, and not significantly contribute to the model.

The final model included average ambient air temperature and the average temperature of the prior 30 days, yet the 3-days and 7-days prior to the sampling date averages were not selected. It is likely that the three- and 7-days averages are not providing the model with unique information because of the focus on photic zone temperature. The upper 2 m of the water column is more responsive to short term temperature changes compared to the whole lake, thus the three- and 7-days temperatures are likely redundant with the sample date temperature. Yet the longer term 30-days average does have an impact on lake photic zone temperature, more specifically, this measures the long-term thermal heating happening at a site. The 30-days average provides us with information about the temperature intensity leading up to the sample date. Sample date, the most important variable to model accuracy, also provides information regarding the seasonal thermal heating of a water body (Wetzel 2001).

To predict lake surface water temperature, other studies have also explored the optimal time interval over which to average ambient air temperature. Jacobsen and Bachman (1974) determined that a period between 10 and 26 days was optimal for their four lakes over 16 years. Matuszek and Shuter (1996) optimal period varied between 5 and 20 days for 14 lakes over 22 years. In a study to predict lake temperature across United Kingdom (UK) lakes, Thrush and Peeler (2013) determined that it was ideal to average temperature over the period of 10 days. It is striking that our model selected a somewhat longer average time period (30 days) than these earlier studies. Given the large spatial extent used for our model input, a longer time interval would be required to account for temperature variability across the continental US.

Using only easily obtainable open-source input data to estimate lake temperature was a primary focus in the creation of this model. Several studies (e.g., Piccolroaz et al., 2018) have used similar metrics (air temperature from downscaled General Circulation Model) with good results (RMSE = 0.5°C) to estimate lake temperature but were applied to a single, or a small number of lakes. Other studies have used more complex models (e.g., General Lake Model (GLM)) to yield good results (RMSE = 1.62°C for the epilimnion) at varying scales (Bruce et al., 2018; Hipsey et al., 2019). However, these models require more complex input variables which are not available for all lakes. Using the NLA data and simple metrics allowed this model to be applicable to any lake within the conterminous US using minimal input data with a comparable RMSE.

Despite being one of the most common measurements collected by limnologists, lake temperature data sets that cover long periods of time uniformly measured for multiple lakes are not readily available. Sharma et al. (2015) have compiled summer lake temperature data for 291 lakes for the period 1985–2009. This may be the largest lake temperature database to date. One of the reasons we chose to model lake photic zone temperature was to develop a database of lake temperatures for the conterminous US. The model we present is shown to be accurate and will allow us to backcast lake temperatures for all of the >300,000 lakes included in National Hydrography Dataset plus for the time period covered by the PRISM climate predictions (1981 to present). This data set will allow us to investigate how photic zone temperatures vary both spatially and temporally across the US. This database is being developed and, when complete, will be made available as an open-source data set.

## Supplementary Material

Supplementary Table S1

## Figures and Tables

**TABLE 1 ∣:** Summary table for selected variables.

Variable	Min	25th	Median	Mean	75th	Max
Date (Day of Year)	152	192	212	212	234	271
Elevation (Meters)	−68.38	193.50	338.24	626.20	695.86	3,639.91
Latitude (Decimal Degrees)	26.07	37.42	41.34	40.71	44.76	48.96
Longitude (Decimal Degrees)	−124.64	−105.14	−94.56	−94.89	−84.77	−67.20
Lake Shoreline Length (km)	0.40	2.00	4.36	24.07	10.55	2,315.20
Lake Area (km^2^)	0.01	0.15	0.48	8.18	1.75	1,674.90
Day of Air Temperature (°C)	3.29	18.17	22.06	21.62	25.52	34.95
Average Air Temperature 30 Day Prior (°C)	5.39	18.80	21.62	21.49	24.59	35.50

## Data Availability

The original data and code presented in the study are included in https://github.com/usepa/lake_photic_zone. Further inquiries can be directed to the corresponding author.
