# Supplementary material for: Predictive Model of Lake Photic Zone Temperature Across the Conterminous United States
Source: Front Environ Sci. Author manuscript; Available in PMC 2021 Dec 8. (PMC8653866; doi:10.3389/fenvs.2021.707874)
Supplement: Supplementary Table S1 [file NIHMS1758100-supplement-Supplementary_Table_S1.docx]

Table S1: Full list of parameters included in initial model. All data and code can be downloaded at github.com/USEPA/lake_photic_zone.git

| **Field** | **Source** | **Included in variable selection**  **process** | **Units** | **Description** |
| --- | --- | --- | --- | --- |
| **year** | lmorpho | Yes | YYYY | year sample of NLA survey (2007 or 2012) |
| **comid** | lmorpho |  | integer | nhdplus common identifier from the lmorpho dataset |
| **nla_id** | nla design |  | character | unique identifiers for the NLA lakes |
| **visit_no** | nla design |  | integer | 1 == first visit to lake; 2 == 2nd visit (a subset) |
| **sample_date** | nla design | Yes | YYYY-MM-DD | date of lake visit |
| **lon_dd** | nla design | Yes | NAD83 dd | longitude of sample location (not exact) |
| **lat_dd** | nla design | Yes | NAD83 dd | latitude of sample location (not exact) |
| **temperature_mean_2m** | nla profile |  | degrees C | mean temperature for depth <= 2 m |
| **temperature_n** | nla profile |  | integer | number of observations used for "temperature_mean_2m" |
| **tmin_dm30:tmin_dm1** | prism |  | degrees C | "tmin" = estimated minimum air temperature; "dm?" == sample_date minus (30:1) days |
| **tmin_d0** | prism |  | degrees C | "tmin" = estimated minimum air temperature for sample_date |
| **tmax_dm30:tmax_dm1** | prism |  | degrees C | "tmax" = estimated maximum air temperature; "dm?" == sample_date minus (30:1) days |
| **tmax_d0** | prism |  | degrees C | "tmax" = estimated maximum air temperature for sample_date |
| **tmean_dm30:tmean_dm1** | prism |  | degrees C | "tmean" = estimated mean air temperature; "dm?" == sample_date minus (30:1) days; calculated as ((tmin_dm? + tmax_dm?) / 2) |
| **tmean_d0** | prism | Yes | degrees C | "tmean" = estimated mean air temperature for sample_date; calculated as ((tmin_d0 + tmax_d0) / 2 |
| **imperv** | NLCD | Yes | percent | percent impervious surface in a 3km buffer around the lake (includes islands); only observations with 100% coverage kept (i.e., lakes with partial NLCD coverage such as border lakes are excluded) |
| **surface_area** | lmorpho | Yes | m^2^ | estimated lake surface area estimated |
| **shoreline_length** | lmorpho | Yes | m | estimated lake shoreline length estimated |
| **shoreline_dev** | lmorpho | Yes | numeric | shoreline development index: calculated from surface_area & shoreline_length |
| **max_length** | lmorpho | Yes | m | estimated maximum length of the lake polygon |
| **max_width** | lmorpho | Yes | m | estimated maximum width of the lake polygon |
| **mean_width** | lmorpho | Yes | m | estimated mean width of the lake polygon |
| **max_depth** | lmorpho | Yes | m | estimated maximum depth |
| **mean_depth** | lmorpho | Yes | m | estimated mean depth |
| **volume** | lmorpho | Yes | m^3^ | estimated total lake volume |
| **elevation** | elevatr | Yes | meters | elevations of the lake |

References:

Hart, E. M., and K. Bell. 2015. Prism: Download data from the oregon prism project.

Hollister, J., and J. Stachelek. 2017. Lakemorpho: Calculating lake morphometry metrics in r. F1000Research 6.

Hollister, J., and Tarak Shah. 2017. Elevatr: Access elevation data from various apis.

Homer, C., Huang, C., Yang, L., Wylie, B. and Coan, M. 2004. Development of a 2001 national land-cover database for the United States. *Photogrammetric Engineering & Remote Sensing*, *70*(7): 829-840.

Homer, C.H., Fry, J.A. and Barnes, C.A. 2012. The national land cover database. *US Geological Survey Fact Sheet*, *3020*(4): 1-4.
